# Global research trends and future frontiers in the immunology of spontaneous abortion: a 25-year scientometric analysis

**DOI:** 10.3389/fimmu.2026.1753732

**Published:** 2026-04-21

**Authors:** Weiyu Qiu, Shiqi Wang, Yanwei Huang, Nuo Xu, Jiesi Yang, Haiwang Wu

**Affiliations:** 1 The Second Affiliated Hospital, Guangzhou Medical University, Guangzhou, China; 2 The First Clinical Medical School of Guangzhou University of Chinese Medicine, Guangzhou University of Chinese Medicine, Guangzhou, China; 3 The Second School of Clinical Medicine , Guangzhou Medical University, Guangzhou, China; 4 Department of Gynecology, The First Affiliated Hospital of Guangzhou University of Chinese Medicine, Guangzhou, China; 5 Guangdong Clinical Research Academy of Chinese Medicine, Guangzhou, China

**Keywords:** citespace, immunology, research trends, scientometrics, spontaneous abortion

## Abstract

**Background:**

The intricate role of the immune system in spontaneous abortion is a critical and rapidly evolving field of reproductive medicine.

**Methods:**

We conducted a scientometric analysis of 4,495 publications on the immunology of spontaneous abortion retrieved from the Web of Science Core Collection for the period 2000–2025. Bibliometric tools, including CiteSpace and HistCite, were used to analyze publication trends, collaboration networks, co-citation patterns, and keyword evolution to identify the intellectual structure and research frontiers.

**Results:**

The annual publication output experienced rapid growth from 2008, peaking in 2022. The American Journal of Reproductive Immunology was the most prolific journal, while China and the USA were the leading countries in scientific collaboration. Co-citation analysis identified foundational works by authors such as Atik RB (2018) and Quenby S (2021). Burst detection analysis revealed a dynamic shift in research hotspots over time, with recent high-strength keywords including “recurrent implantation failure” (strength: 15.87), “chronic endometritis” (13.4), and “maternal-fetal interface” (10.35). Timeline and cluster analyses further pinpointed the field’s evolution from foundational topics like “tolerance” and “antiphospholipid syndrome” to current emerging frontiers. These new frontiers are concentrated in distinct clusters, prominently including #0 recurrent pregnancy loss, #2 maternal-fetal interface, #11 chronic endometritis, and #12 COVID-19. Alluvial flow visualization confirmed that themes related to “regulatory_t_cells” and therapeutic interventions at the “maternal-fetal_interface” represent major developing research streams.

**Conclusion:**

This study comprehensively maps the historical landscape and dynamic evolution of research on immunology in spontaneous abortion, identifying key emerging frontiers that may represent important directions for future investigation in the field.

## 1 Introduction

Pregnancy presents a profound immunological paradox: the maternal immune system must tolerate the semi-allogeneic fetus, which expresses paternal antigens, while simultaneously maintaining robust defense against pathogenic threats (1, 2). The establishment and maintenance of this delicate state of maternal-fetal immune tolerance is a fundamental prerequisite for a successful pregnancy (3). However, this intricate balance is fragile, and its disruption can lead to devastating clinical outcomes, the most common of which is spontaneous abortion (SA) (4). Globally, an estimated 23 million miscarriages occur each year, with the overall risk in clinically confirmed pregnancies reaching 15.3%, imposing a significant physiological, psychological, and economic burden on women and their families worldwide (5, 6). When pregnancy loss is recurrent, termed recurrent pregnancy loss (RPL), it not only amplifies this trauma but is also recognized as a sentinel event for future obstetric complications and long-term health risks, underscoring the urgent need for a deeper understanding of its etiology (7, 8). In this study, Spontaneous Abortion (SA)—often used interchangeably with the lay term Miscarriage—is defined as the involuntary loss of a conceptus before 20–24 weeks of gestation. Conversely, Recurrent Pregnancy Loss (RPL), also referred to as Recurrent Spontaneous Abortion (RSA), is a distinct clinical entity traditionally defined by two or more (ASRM) or three or more (ESHRE) consecutive pregnancy losses. While SA may involve isolated genetic errors, RPL is frequently underpinned by complex maternal-fetal immunological dysregulation, which serves as the primary focus of this scientometric analysis.

Over the past two decades, propelled by rapid advances in immunological theory and technology, a consensus has emerged that a substantial proportion of unexplained SA is fundamentally an immune-mediated disorder (9, 10). Research has progressively shifted from conventional anatomical, endocrine, and genetic etiologies toward the complex immune microenvironment of the maternal-fetal interface (11, 12). This unique anatomical site, where maternal and fetal tissues are in direct apposition, hosts a specialized milieu of immune cells whose composition and functional state are decisive for pregnancy success or failure (13). In a healthy gestation, this interface is enriched with immunoregulatory cell populations, including regulatory T cells (Tregs) (14, 15), regulatory B cells (Bregs) (16), M2-polarized macrophages (17), and uterine natural killer (uNK) cells with a unique, less cytotoxic phenotype (18). Through the secretion of anti-inflammatory cytokines such as interleukin-10 (IL-10) and transforming growth factor-β (TGF-β), and the expression of inhibitory molecules, these cells collaboratively construct an immune-privileged environment that actively suppresses aggressive immune responses against the fetus while promoting essential processes like trophoblast invasion and vascular remodeling (19–21).

In patients experiencing spontaneous abortion, however, this finely tuned regulatory network is frequently compromised. A vast body of clinical and basic research confirms that a shift toward a pro-inflammatory state at the maternal-fetal interface is a central mechanism of pregnancy failure (22). For instance, a skewed balance favoring T helper 1 (Th1) and T helper 17 (Th17) cells leads to the overproduction of inflammatory cytokines like interferon-γ (IFN-γ), tumor necrosis factor-α (TNF-α), and interleukin-17 (IL-17), which exert direct and indirect damaging effects on placental tissues (23, 24). Concurrently, a reduction in the number or function of Tregscripples their capacity to restrain effector T cells, leading to a breakdown in maternal tolerance (25). Furthermore, the activation of more cytotoxic natural killer (NK) cell subsets in both peripheral blood and the endometrium, along with incompatibilities between maternal killer-cell immunoglobulin-like receptors (KIR) and fetal human leukocyte antigen-C (HLA-C), have been identified as significant contributors to pregnancy loss (18, 26). Beyond cellular dysregulation, abnormalities in humoral immunity, particularly the presence of autoantibodies in conditions like Antiphospholipid Syndrome (APS), can induce miscarriage by activating complement, promoting thrombosis, and directly impairing trophoblast function (27, 28).

In recent years, the application of high-throughput technologies such as single-cell RNA sequencing and spatial transcriptomics has propelled our understanding of the maternal-fetal dialogue to unprecedented depths (29). These approaches have not only unveiled novel immune cell subtypes and their specialized roles in establishing pregnancy but have also cemented the “maternal-fetal interface” as a dominant research hotspot. Concurrently, clinical entities previously overlooked have gained prominence. Chronic endometritis(CE), a persistent low-grade inflammation of the endometrium, has been strongly associated with both RPL and recurrent implantation failure(RIF), likely through mechanisms involving aberrant immune cell infiltration and impaired endometrial receptivity (30). Moreover, global public health crises, notably the coronavirus disease 2019 (COVID-19) pandemic, have offered new paradigms for investigation. Emerging evidence suggests that severe acute respiratory syndrome coronavirus 2 (SARS-CoV-2) infection can precipitate adverse pregnancy outcomes by triggering a systemic maternal inflammatory response, establishing “COVID-19” as a critical and emerging area of inquiry within this field (31).

This explosive growth in knowledge and the continuous differentiation of research themes have led to a dramatic increase in the volume of scientific literature on the immunology of spontaneous abortion, particularly since 2008. While traditional narrative reviews have been invaluable for summarizing specific topics, they are often subject to author bias and cannot provide a comprehensive, objective map of the entire field’s intellectual structure, evolutionary trajectory, and future frontiers. In the face of this data deluge, researchers require a more macroscopic, data-driven methodology to systematically chart the intellectual foundations and dynamic evolution of the discipline. Scientometrics, a quantitative approach to literature analysis, employs mathematical and statistical tools to mine large bibliographic datasets, revealing collaboration networks, foundational works, thematic evolution, and emerging hotspots, thereby offering critical insights for researchers and policymakers (32, 33).

Therefore, this study employs scientometric tools, including CiteSpace and HistCite, to conduct the first comprehensive, large-scale visualization analysis of 4,495 publications on the immunology of spontaneous abortion retrieved from the Web of Science Core Collection from 2000 to 2025. Our objective is to: (1) delineate the historical trajectory and overall publication trends of the field over the past 25 years; (2) identify the most influential countries, institutions, authors, and publications that form its intellectual base; (3) dynamically track the evolution of research hotspots through keyword co-occurrence, cluster analysis, and burst detection; and (4) identify current research frontiers and highlight potentially emerging directions based on recent thematic patterns. Through this systematic, panoramic assessment, we aim to provide the scientific community with a clear intellectual map to navigate this complex field and to provide a reference framework for future basic and clinical research.

## 2 Methods

### 2.1 Data acquisition and statistical collation

The Web of Science Core Collection (WoSCC) includes a large number of high-impact academic journals and is widely recognized as an authoritative source for bibliometric research. In this study, WoSCC was selected as the primary database. This exclusive choice was methodologically necessary, as the advanced co-citation and historiographic algorithms employed by CiteSpace and HistCite rely on the highly standardized and complete citation metadata that only WoSCC provides. Merging multiple databases would introduce formatting inconsistencies and distort citation-based metrics.

On August 1, 2025, we conducted a systematic literature search covering publications from January 1, 2000 to August 1, 2025, with data spanning over 25 years. Given the interdisciplinary nature of research on the immunology of spontaneous abortion, both the Science Citation Index Expanded (SCI-EXPANDED) and the Social Sciences Citation Index (SSCI) were included in the search scope. In addition to basic and clinical biomedical research, this field also intersects with perspectives from public health, health behavior, and health policy. Including the SSCI therefore allowed us to more comprehensively capture the knowledge structure and citation relationships across these areas.

The search strategy was constructed using topic terms (TS) as follows: TS=(“Abortion, Spontaneous” OR “Abortions, Spontaneous” OR “Spontaneous Abortions” OR “Spontaneous Abortion” OR “Abortion, Tubal” OR “Abortions, Tubal” OR “Tubal Abortion*” OR “Miscarriage*” OR “Early Pregnancy Loss*” OR “Loss*, Early Pregnancy” OR “Pregnancy Loss*, Early”) AND TS=(immune OR immun* OR immunotherapy).This search was intentionally designed to cover the full spectrum of pregnancy loss. By including both general terms (e.g., “miscarriage”) and more specific clinical entities (e.g., “recurrent pregnancy loss”), we aimed to capture the shared immunological mechanisms underlying both sporadic and recurrent cases.

The inclusion and exclusion criteria were as follows. Inclusion criteria: (1) peer-reviewed original articles and reviews; (2) English-language publications; and (3) a publication timeframe from January 1, 2000, to August 1, 2025. Exclusion criteria: (1) meeting abstracts, editorial materials, letters, and book chapters; (2) non-English literature; and (3) duplicated/retracted records.

All retrieved records were downloaded and exported as plain text files with the “Full Record and Cited References” option selected to preserve complete bibliographic information for subsequent analysis. After screening and deduplication, a total of 4,495 unique publications were included in the final dataset for bibliometric analysis.

### 2.2 Bibliometric analysis tools

#### 2.2.1 CiteSpace

Co-occurrence Network Analysis. Scientific partnership, defined as the simultaneous appearance of multiple authors, institutions, or countries/regions within a single publication, is a cornerstone of modern research. The analysis of such scientific collaboration networks can effectively reveal the research landscape of a specific domain, reflecting its structure from the dimensions of individual researchers, research institutions, and international participation. When a dataset of publications is imported into the CiteSpace software (Version 6.2.R4), these collaborative relationships and core scientific concepts can be rendered as a visualized co-occurrence network.

In these visualizations, CiteSpace employs a system of color-coded nodes and edges to construct a merged network, with colors assigned based on the year of appearance in the dataset. The color of an edge (the link between nodes) signifies the year when the co-occurrence link was first established. The nodes are composed of concentric “tree rings,” where the thickness of each ring is proportional to the co-occurrence count in a given year. The presence of a red ring indicates a “citation burst” for that year, signifying a sharp increase in the frequency of citations. A purple ring is used to denote the degree of a node’s betweenness centrality; nodes with high betweenness centrality are considered significant as they often serve as critical connectors bridging disparate clusters of research.

Burst Detection. As conceptualized by Jon Kleinberg, document streams, such as emails or scholarly articles, exhibit thematic focuses that are prominent for a certain period before diminishing. Such temporal shifts in topics can be identified using specific text data-mining algorithms, represented as periods of “activity bursts.” Building upon Kleinberg’s algorithm, Chen et al. adapted this concept to bibliometrics, defining a citation burst as an indicator of a particularly active or emerging research theme. A citation burst represents a significant surge in citations for a particular entity (e.g., a keyword or reference) that may persist for one or more years. CiteSpace facilitates burst detection for subject categories, keywords, and individual references. The occurrence of a citation burst indicates that a particular subject, keyword, or reference has garnered intense and focused attention from the scientific community.

Cluster Analysis. To delineate the conceptual structure of a research field, CiteSpace integrates three distinct clustering algorithms that operate on titles, abstracts, and keywords. This functionality categorizes publications into discrete conceptual clusters, each representing a different salient feature of the research domain. The resulting cluster map, configured according to the time-slicing parameters, illustrates the evolution of these conceptual clusters over different periods. Furthermore, the timeline view provides a clear visualization of the temporal trajectory of each cluster—its rise and fall in prominence—and highlights the key nodes that connect it with other clusters. For cluster identification, we utilized the Log-Likelihood Ratio (LLR) algorithm to extract label terms from titles, abstracts, and keywords. The network’s structural robustness was validated using Modularity (Q value) and Mean Silhouette (S value), where Q > 0.3 indicates a significant structure and S > 0.7 signifies high cluster credibility.

Analytical Procedure. The DATA dataset pertaining to immunity in spontaneous abortion was imported into CiteSpace (v. 6.2.R4). The primary parameters were configured as follows: Time Slicing was set from 2000 to 2025 (inclusive of the partial year up to August), with an interval of 1 year per slice. For the generation of collaboration networks, the node types selected were “Country,” “Institution,” or “Author,” respectively. Term sources were selected from “Title,” “Abstract,” “Author Keywords (DE),” and “Keywords Plus (ID).” All other settings were maintained at their default values. The software automatically generated the initial knowledge graphs, which were subsequently adjusted manually to enhance clarity and aesthetic presentation. For the analysis of keyword evolution, a similar methodology was employed, but with “Keyword” selected as the node type and the time period segmented into four distinct slices: 2000–2006, 2007–2013, 2014–2019, and 2020–2025. To generate the citation timeline graph, “Reference” was chosen as the node type, and the “Timeline View” option was selected under the “Layout” tab in the control panel. Finally, burst detection maps for keywords, categories, or references were generated by selecting the “Burstness” tab in the control panel and executing the “View” command.

#### 2.2.2 HistCite

To identify the most influential publications within the research field, we utilized HistCite Pro 2.1 software. Each publication can be regarded as a node in the citation network, and citation frequency reflects its relative visibility within the dataset. Consequently, highly cited works can be identified more readily in the historiograph. HistCite Pro 2.1 constructs a historiograph that maps these citation relationships, enabling the rapid identification of landmark publications. The software scores articles using two primary metrics: the Local Citation Score (LCS), which represents the citation frequency of a paper within the imported dataset, and the Global Citation Score (GCS), which reflects the total citation count in the broader WoSCC database. The DATA dataset (n=4,495) was imported into HistCite Pro 2.1, and the record limit for the analysis was set to the top 30. All other settings remained at their default values. The “Make Graph” function was then executed to generate a chronological map of the field, thereby facilitating the swift localization of pivotal literature.

#### 2.2.3 The alluvial generator

To elucidate the temporal evolution and thematic shifts within the co-occurrence network, alluvial flow diagrams were generated. This visualization technique is specifically designed to illustrate dynamic patterns in evolving networks over time. The workflow involved using CiteSpace to generate a series of individual co-occurrence keyword networks for distinct time slices. These network files were then exported from CiteSpace and loaded into the online Alluvial Generator application (available at http://www.mapequation.org/apps/AlluvialGenerator.html).

In this model, each keyword is treated as a node. Nodes are grouped into clusters at each time slice, with each cluster representing a module or research theme. Over successive time slices, nodes may split, merge, or transition between modules, forming new thematic groupings. The flow diagram visualizes this process, where the composition of modules in a later period is formed by the intersection of nodes from preceding periods, thereby clearly mapping the dynamic trajectory of research topics.

#### 2.2.4 Data visualization in R

**Figure 1:**
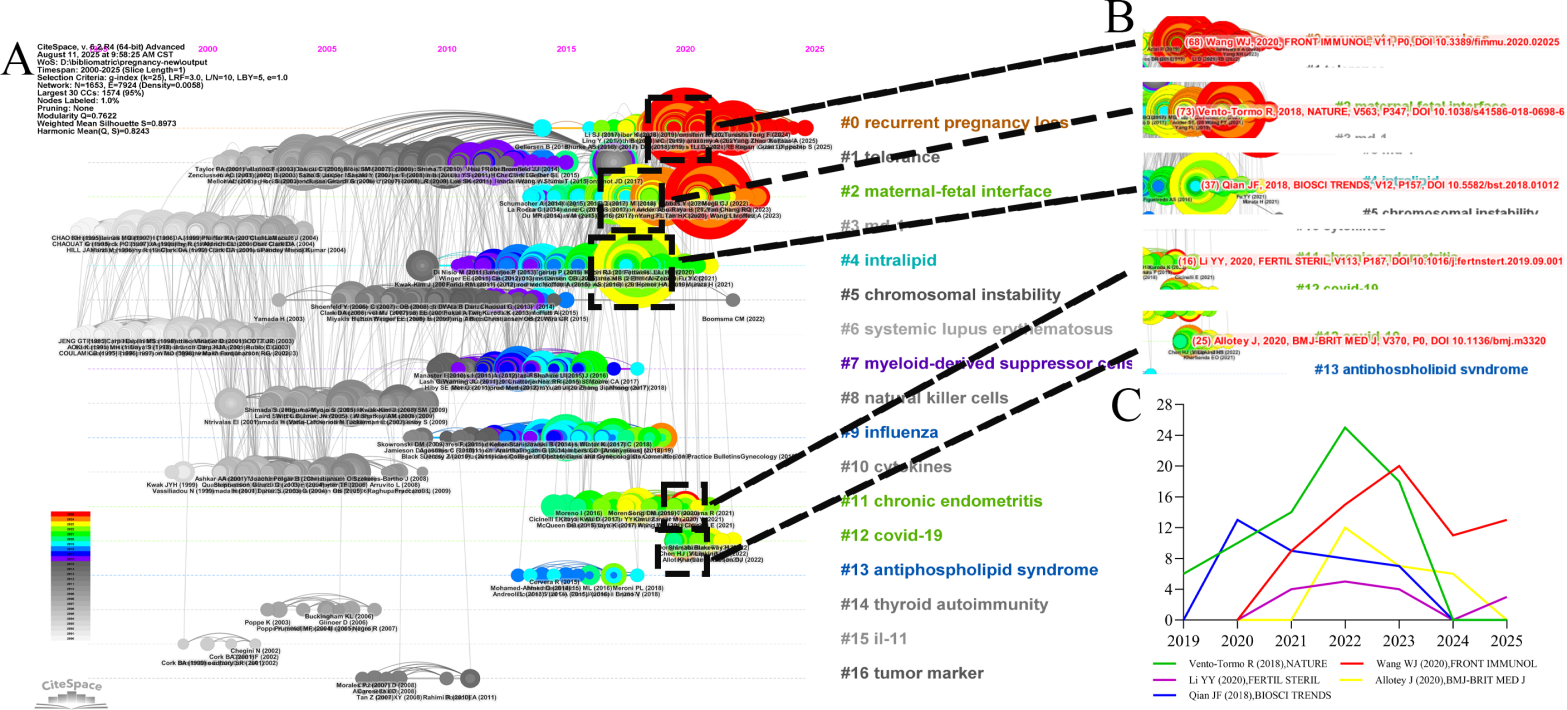
Recent citation trends of influential publications. **(A)** Timeline of Co-cited References. **(B)** Representative Breakthrough Publications in Emerging and Core Research Themes. **(C)** Annual citation frequency from 2019 to 2025 for a selection of highly cited papers published between 2018 and 2020. The trajectories illustrate the varying temporal impact and sustained influence of key studies in shaping contemporary spontaneous abortion research.

The donut chart presented in **Figure 1** was generated using the R statistical computing environment (version 4.2.2). Specifically, the chart was constructed utilizing the geom_bar function within the ggplot2 package (version 3.4.4) to visualize proportional data.

## 3 Results

### 3.1 The historical features of the literature on immunology in spontaneous abortion

#### 3.1.1 Distribution of publications

**Figure 2:**
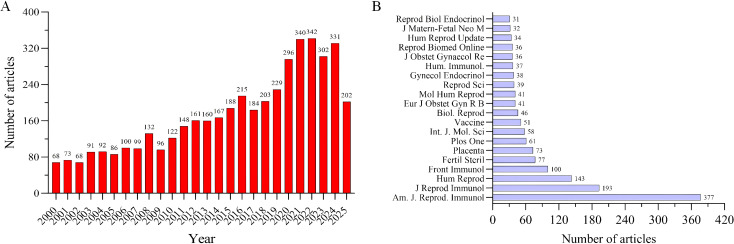
Publication trends and key journals in spontaneous abortion research. **(A)** The top 20 most productive academic journals publishing on miscarriage. The publication count for the year 2025 represents a partial year, encompassing data retrieved from January 1 to August 1, 2025. **(B)** The annual number of publications on miscarriage from 2002 to 2023, showing a consistent growth in research output over time. Data sourced from the Web of Science Core Collection (WoSCC).

Our systematic search retrieved 4,495 publications on immunology in spontaneous abortion, involving 19,445 authors from 4,665 institutions and published in 1,110 journals across 139 scientific categories (**Table 1**). As shown in **Figure 2A**, annual publication output remained relatively low from 2000 to 2007, increased markedly from 2008 to 2022, peaked in 2022, and then declined thereafter. The American Journal of Reproductive Immunology was the most prolific outlet, contributing 377 articles, followed by the Journal of Reproductive Immunology (193 articles) and Human Reproduction (143 articles). **Figure 2B** lists the top 20 journals by publication volume, providing a reference for researchers considering venues for their work.

**Table 1:** Basic information on the distribution of the publications.

Categories	Publication	Articles	Review	Authors	Institutions	Journals	Subject categories
Amount	4495	3586	909	19445	4663	1110	139

#### 3.1.2 The intellectual foundation of research on immunology in spontaneous abortion

**Figure 3:**
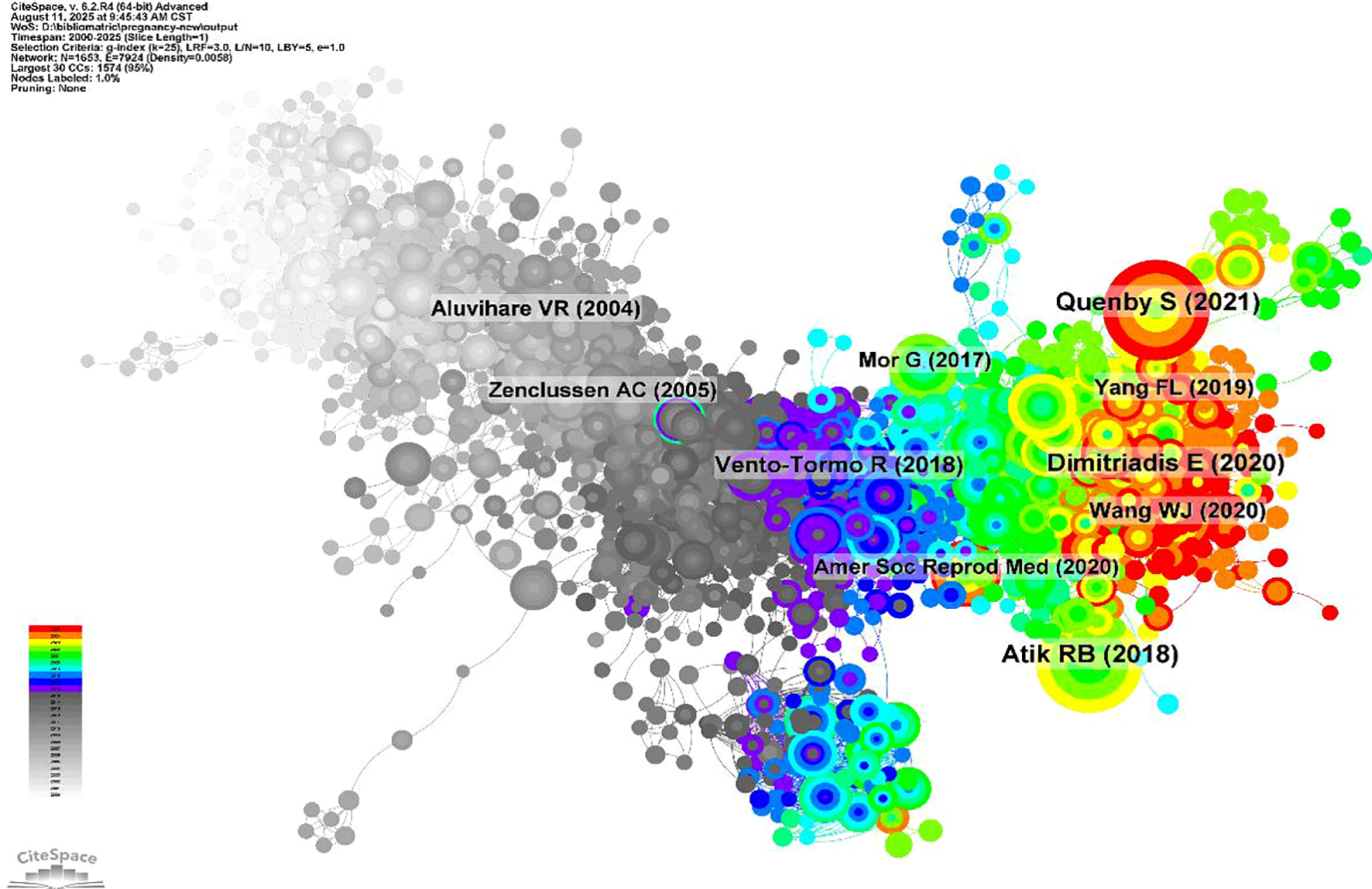
Citation co-citation network of spontaneous abortion research literature. The network illustrates the relationships between key publications, with node colors representing their publication year from 2000 (white) to 2025 (red). The structure reveals the foundational core of the field and the subsequent emergence and evolution of distinct research fronts over time.

A co-citation analysis reveals the intellectual structure of the field over the past two decades (**Figure 3**). The network comprises 1,653 nodes and 7,924 links, indicating extensive interconnections among publications. Foundational works from the early period contributed substantially to the development of the field, whereas studies from the intermediate and recent periods showed increasing diversification and the gradual formation of more focused clusters. Among these publications, ten articles showed particularly high co-citation frequencies, including Atik RB (2018), Quenby S (2021), Dimitriadis E (2020), Vento-Tormo R (2018), Wang WJ (2020), Yang FL (2019), Aluvihare VR (2004), Zenclussen AC (2005), Amer Soc Reprod Med (2020), and Mor G (2017). These highly co-cited references provide an important basis for the subsequent reference timeline analysis. Additionally, a citation historiograph was constructed using HisCite Pro 2.1. Landmark articles identified through this method are highlighted in **Table 2**, with the top three being “Decidual and peripheral blood CD4+CD25+ regulatory T cells in early pregnancy subjects and spontaneous abortion cases”, “Abnormal T-cell reactivity against paternal antigens in spontaneous abortion”, and “Adoptive transfer of pregnancy-induced CD4+CD25+ T regulatory cells prevents fetal rejection in a murine abortion model & Th1/Th2/Th17 and Regulatory T-Cell Paradigm in Pregnancy.” These articles are particularly notable for their high Local Citation Scores (LCS) and Global Citation Scores (GCS), indicating their significant impact both within the dataset of this study and in the broader scientific community.

**Table 2:** The information of the top 30 literature sorted by LCS and GCS score.

NO.	Article information	Journal	LCS	GCS
342	Decidual and peripheral blood CD4+CD25+ regulatory T cells in early pregnancy subjects and spontaneous abortion cases	MOL HUM REPROD	263	630
413	Abnormal T-cell reactivity against paternal antigens in spontaneous abortion -: Adoptive transfer of pregnancy-induced CD4+CD25+ T regulatory cells prevents fetal rejection in a murine abortion model	AM J PATHOL	226	471
955	Th1/Th2/Th17 and Regulatory T-Cell Paradigm in Pregnancy	AM J REPROD IMMUNOL	186	902
932	Increased prevalence of T helper 17 (Th17) cells in peripheral blood and decidua in unexplained recurrent spontaneous abortion patients	J REPROD IMMUNOL	152	292
20	Cytokine production by maternal lymphocytes during normal human pregnancy and in unexplained recurrent spontaneous abortion	HUM REPROD	132	321
1565	Natural killer cells in female infertility and recurrent miscarriage: a systematic review and meta-analysis	HUM REPROD UPDATE	117	218
875	Regulatory T-cells and immune tolerance in pregnancy: a new target for infertility treatment?	HUM REPROD UPDATE	106	383
530	Primary unexplained infertility is associated with reduced expression of the T-regulatory cell transcription factor Foxp3 in endometrial tissue	MOL HUM REPROD	96	269
233	A review of immune cells and molecules in women with recurrent miscarriage	HUM REPROD UPDATE	94	464
1405	Fetomaternal immune cross-talk and its consequences for maternal and offspring’s health	NAT MED	92	223
729	Association of maternal killer-cell immunoglobulin-like receptors and parental HLA-C genotypes with recurrent miscarriage	HUM REPROD	85	244
961	Regulatory T cells are necessary for implantation and maintenance of early pregnancy but not late pregnancy in allogeneic mice	J REPROD IMMUNOL	82	265
16	Embryonic karyotype of abortuses in relation to the number of previous miscarriages	FERTIL STERIL	81	359
159	A randomized, double-blind, placebo-controlled trial of intravenous immunoglobulin in the prevention of recurrent miscarriage: evidence for a therapeutic effect in women with secondary recurrent miscarriage	HUM REPROD	78	125
96	Status of peripheral blood natural killer cells in women with recurrent spontaneous abortions and infertility of unknown aetiology	HUM REPROD	76	145
127	Th1 and Th2 cytokine profiles in recurrent aborters with successful pregnancy and with subsequent abortions	HUM REPROD	75	220
917	Detailed analysis of peripheral blood natural killer (NK) cells in women with recurrent miscarriage	HUM REPROD	75	122
591	Use of intravenous immunoglobulin for treatment of recurrent miscarriage: a systematic review	BJOG-INT J OBSTET GY	71	100
31	Peripheral natural killer cytotoxicity and CD56posCD16pos cells increase during early pregnancy in women with a history of recurrent spontaneous abortion	HUM REPROD	71	106
1113	Natural killer cells and pregnancy outcomes in women with recurrent miscarriage and infertility: a systematic review	HUM REPROD	70	398
897	The CD4+CD25bright regulatory T cells and CTLA-4 expression in peripheral and decidual lymphocytes are down-regulated in human miscarriage	CLIN IMMUNOL	69	190
1079	Study on the Relationship Between Th17 Cells and Unexplained Recurrent Spontaneous Abortion	AM J REPROD IMMUNOL	69	140
635	Proportion of peripheral blood and decidual CD4+ CD25bright regulatory T cells in pre-eclampsia	CLIN EXP IMMUNOL	68	164
820	Uterine natural killer cells and angiogenesis in recurrent reproductive failure	HUM REPROD	68	291
1009	Maternal activating KIRs protect against human reproductive failure mediated by fetal HLA-C2	J CLIN INVEST	67	122
990	Intravenous immunoglobulin and idiopathic secondary recurrent miscarriage: a multicentered randomized placebo-controlled trial	HUM REPROD	67	133
463	Prednisolone reduces preconceptual endometrial natural killer cells in women with recurrent miscarriage	FERTIL STERIL	67	311
559	Complement activation induces dysregulation of angiogenic factors and causes fetal rejection and growth restriction	J EXP MED	65	141
1078	Cytokines in recurrent pregnancy loss	CLIN CHIM ACTA	64	417
378	Heparin prevents antiphospholipid antibody-induced fetal loss by inhibiting complement activation	NAT MED	64	128

#### 3.1.3 Scientific cooperation

**Figure 4:**
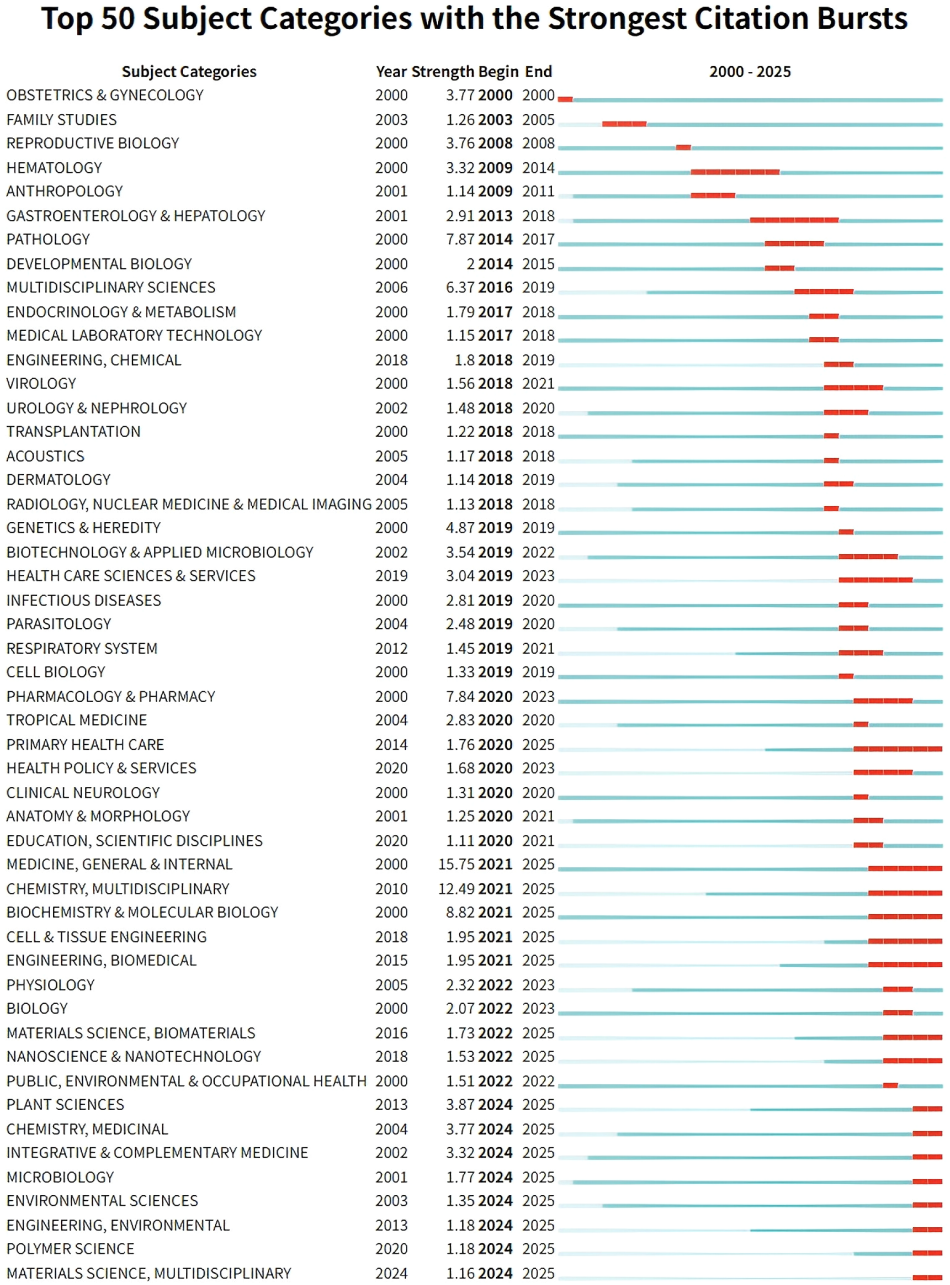
The top 50 keywords with the strongest citation bursts in spontaneous abortion research from 2000 to 2025. The red segments on the timeline indicate the duration of a keyword’s burst period, reflecting intervals when it attracted significant scholarly attention. The ‘Strength’ value quantifies the intensity of each burst. The data illustrates the dynamic shift in research hotspots from basic immunology to the intricate mechanisms of endometrial receptivity and recurrent reproductive failure.

**Figure 5:**
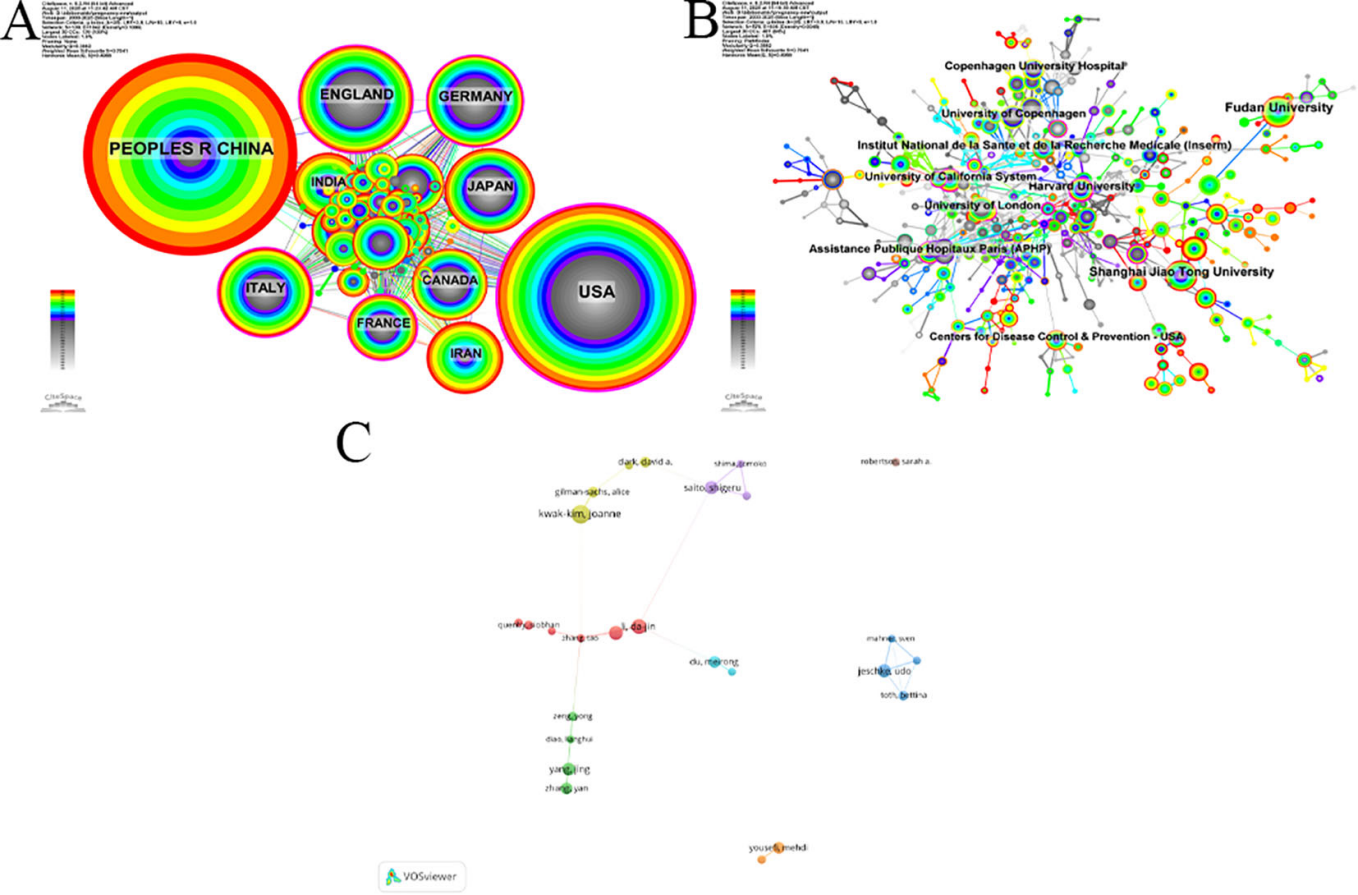
Scientific cooperation networks in spontaneous abortion research. **(A)** Network of country-level collaborations, with node size representing the volume of publications. **(B)** Network of institutional collaborations, showing both international and domestic partnerships. **(C)** Network of author collaborations, highlighting major research teams.

As shown in **Figure 4**, the dense networks, characterized by numerous nodes and links, signify robust scientific collaboration across the levels of countries, institutions, and authors. The international collaboration network consists of 139 nodes and 1,042 links, with the most prominent nodes being China, the USA, the UK, Germany, and Italy (**Figure 5A**). The institutional collaboration network contains 578 nodes and 808 links, with Fudan University, Shanghai Jiao Tong University, Harvard University, and the Institut National de la Sante et de la Recherche Medicale (Inserm) representing the largest nodes (**Figure 5B**). The author collaboration network is presented in **Figure 5C**. Kwak-Kim, Joanne; Li, Da-Jin; Li, Ming-Qing; Jeschke, Udo; and Saito, Shigeru are among the most prolific authors in this domain, and the dense connections between nodes reflect extensive research partnerships.

### 3.2 Variation of the most active topics

#### 3.2.1 Subject category burst

Between 2000 and 2025, a total of 101 out of 139 relevant subject categories experienced citation bursts. **Figure 4** displays the top 50 categories with the highest burst strength during different periods. The subject category MEDICINE, GENERAL & INTERNAL exhibited the highest burst strength (15.75) during its burst period from 2021 to 2025. Notably, bursting categories became increasingly diverse over time, reflecting the increasingly multidisciplinary nature of the field. Furthermore, 20 subject categories remained in burst status into the most recent period (**Supplementary Table 2**), with the top three being MEDICINE, GENERAL & INTERNAL, CHEMISTRY, MULTIDISCIPLINARY, and BIOCHEMISTRY & MOLECULAR BIOLOGY.

#### 3.2.2 Keywords burst

**Figure 6:**
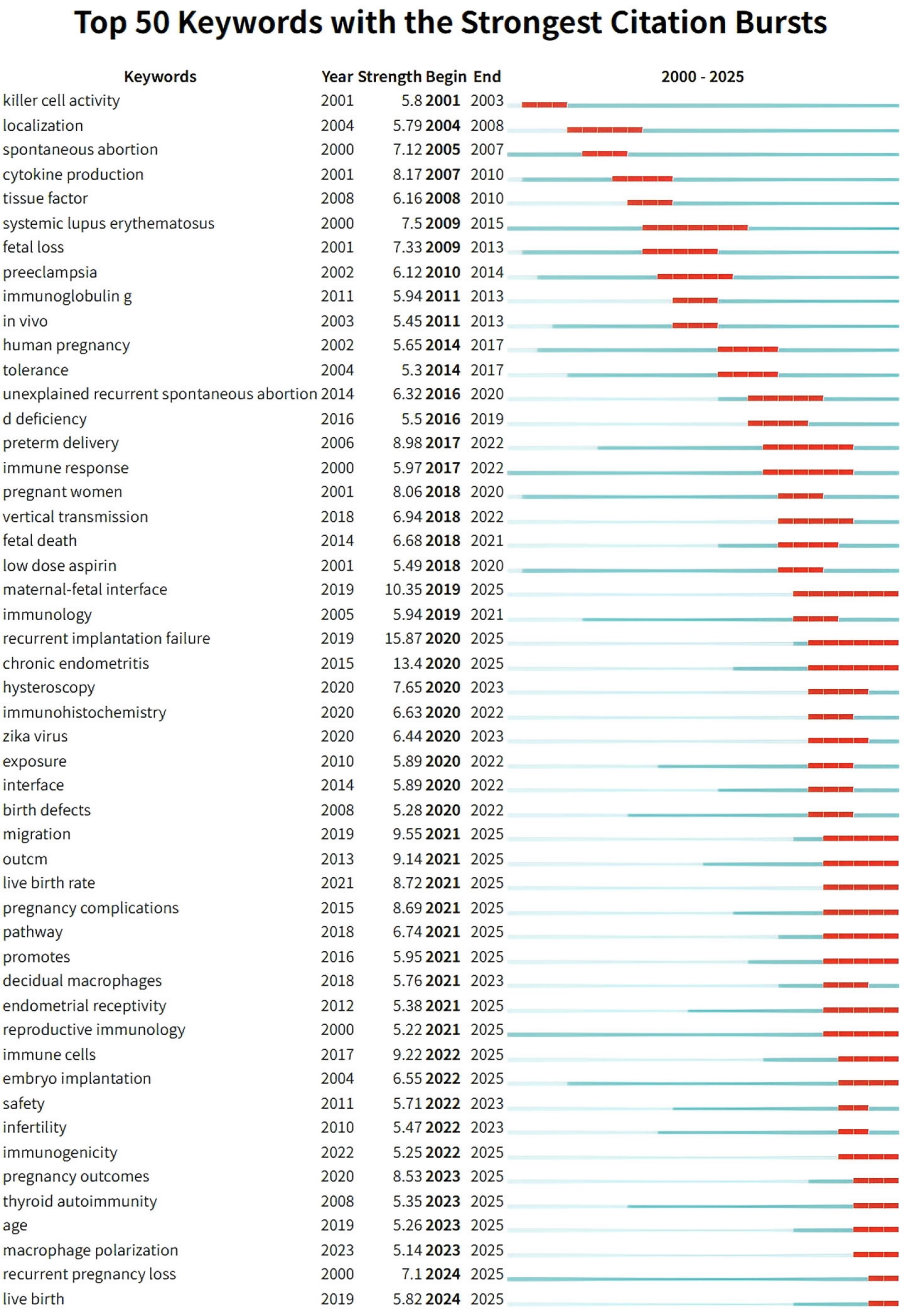
Timeline of the top 50 keywords with the strongest citation bursts in spontaneous abortion research (2000-2025). The red segments indicate the duration of a keyword’s burst period, representing intervals of intense scholarly interest. The ‘Strength’ value quantifies the intensity of each burst. The progression from basic immunology to the cellular mechanisms of the maternal-fetal interface and refined clinical phenotypes is clearly demonstrated.

Keyword burst detection further revealed the most active research topics from 2000 to 2025. Among 755 keywords that experienced bursts at various points, the top 50 with the greatest burst strength are shown in **Figure 6**. The keyword “recurrent implantation failure” had the highest burst strength (15.87) from 2020 to 2025. This was followed by “maternal-fetal interface” (strength: 10.35, duration: 2019–2025) and “migration” (strength: 9.55, duration: 2021–2025). We gave special attention to the 20 keywords whose bursts remain active into 2025, as they may reflect topics of sustained current interest or potentially emerging directions in the field. Key examples include “recurrent implantation failure” (strength: 15.87, 2020–2025), “chronic endometritis” (strength: 13.4, 2020–2025), “maternal-fetal interface” (strength: 10.35, 2019–2025), and “migration” (strength: 9.55, 2021–2025) (**Supplementary Table 2**).

#### 3.2.3 Reference burst

A total of 510 publications were identified as having citation bursts. **Table 3** lists the top 30 references with the strongest citation bursts between 2000 and 2025.

**Table 3:** The references with citation bursts at different period.

References	Year	Strength	Begin	End	2004 - 2025
Raghupathy R, 1997, IMMUNOL TODAY, V18, P478, DOI 10.1016/S0167-5699(97)01127-4, DOI	1997	18.94	**2000**	2002	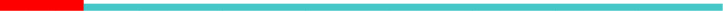
Piccinni MP, 1998, NAT MED, V4, P1020, DOI 10.1038/2006, DOI	1998	18.94	**2000**	2003	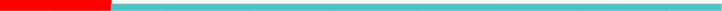
Clark DA, 1999, AM J REPROD IMMUNOL, V41, P5	1999	16.49	**2001**	2004	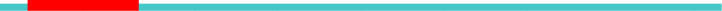
Aluvihare VR, 2004, NAT IMMUNOL, V5, P266, DOI 10.1038/ni1037, DOI	2004	31	**2005**	2009	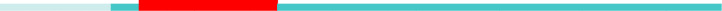
Zenclussen AC, 2005, AM J PATHOL, V166, P811, DOI 10.1016/S0002-9440(10)62302-4, DOI	2005	27.91	**2005**	2010	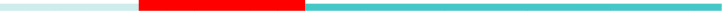
Sasaki Y, 2004, MOL HUM REPROD, V10, P347, DOI 10.1093/molehr/gah044, DOI	2004	19.89	**2005**	2009	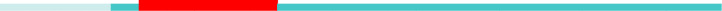
Hanna J, 2006, NAT MED, V12, P1065, DOI 10.1038/nm1452, DOI	2006	21.9	**2007**	2011	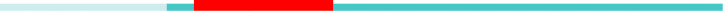
Wang WJ, 2010, J REPROD IMMUNOL, V84, P164, DOI 10.1016/j.jri.2009.12.003, DOI	2010	23.92	**2010**	2015	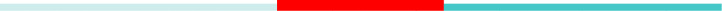
Guerin LR, 2009, HUM REPROD UPDATE, V15, P517, DOI 10.1093/humupd/dmp004, DOI	2009	19.33	**2010**	2014	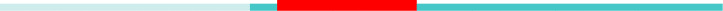
Saito S, 2010, AM J REPROD IMMUNOL, V63, P601, DOI 10.1111/j.1600-0897.2010.00852.x, DOI	2010	22.65	**2011**	2015	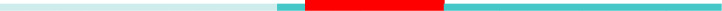
Samstein RM, 2012, CELL, V150, P29, DOI 10.1016/j.cell.2012.05.031, DOI	2012	20.54	**2013**	2016	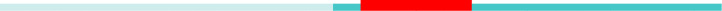
Erlebacher A, 2013, ANNU REV IMMUNOL, V31, P387, DOI 10.1146/annurev-immunol-032712-100003, DOI	2013	20.2	**2014**	2018	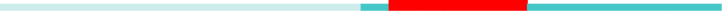
Seshadri S, 2014, HUM REPROD UPDATE, V20, P429, DOI 10.1093/humupd/dmt056, DOI	2014	17.15	**2015**	2019	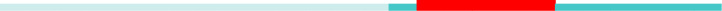
Figueiredo AS, 2016, IMMUNOLOGY, V148, P13, DOI 10.1111/imm.12595, DOI	2016	17.07	**2017**	2021	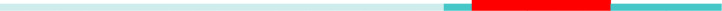
Mor G, 2017, NAT REV IMMUNOL, V17, P469, DOI 10.1038/nri.2017.64, DOI	2017	21.36	**2018**	2022	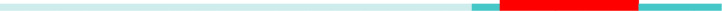
Vento-Tormo R, 2018, NATURE, V563, P347, DOI 10.1038/s41586-018-0698-6, DOI	2018	26.87	**2019**	2023	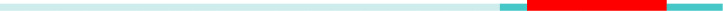
El Hachem H, 2017, INT J WOMENS HEALTH, V9, P331, DOI 10.2147/IJWH.S100817, DOI	2017	17.05	**2019**	2022	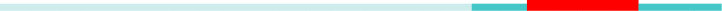
Liu S, 2017, J REPROD IMMUNOL, V124, P44, DOI 10.1016/j.jri.2017.10.045, DOI	2017	16.62	**2019**	2022	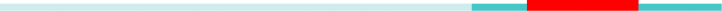
Atik RB, 2018, HUM REPROD OPEN, V2018, P0, DOI 10.1093/hropen/hoy004, DOI	2018	48.98	**2020**	2023	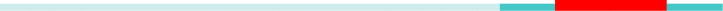
Robertson SA, 2018, J CLIN INVEST, V128, P4224, DOI 10.1172/JCI122182, DOI	2018	19.15	**2020**	2023	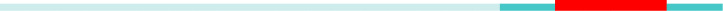
Wang WJ, 2020, FRONT IMMUNOL, V11, P0, DOI 10.3389/fimmu.2020.02025, DOI	2020	23.83	**2021**	2025	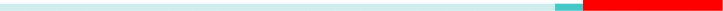
Yang FL, 2019, FRONT IMMUNOL, V10, P0, DOI 10.3389/fimmu.2019.02317, DOI	2019	22.92	**2021**	2025	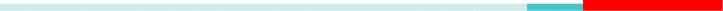
Amer Soc Reprod Med, 2020, FERTIL STERIL, V113, P533, DOI 10.1016/j.fertnstert.2019.11.025, DOI	2020	18.54	**2021**	2025	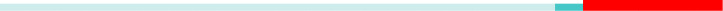
Ander SE, 2019, SCI IMMUNOL, V4, P0, DOI 10.1126/sciimmunol.aat6114, DOI	2019	18.05	**2021**	2025	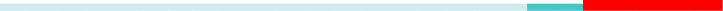
Ticconi C, 2019, INT J MOL SCI, V20, P0, DOI 10.3390/ijms20215332, DOI	2019	16.57	**2021**	2025	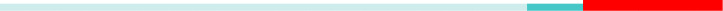
Quenby S, 2021, LANCET, V397, P1658, DOI 10.1016/S0140-6736(21)00682-6, DOI	2021	50.88	**2022**	2025	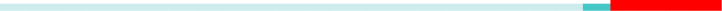
Dimitriadis E, 2020, NAT REV DIS PRIMERS, V6, P0, DOI 10.1038/s41572-020-00228-z, DOI	2020	43.05	**2022**	2025	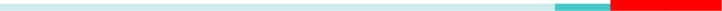
Li D, 2021, REPROD SCI, V28, P3303, DOI 10.1007/s43032-021-00599-y, DOI	2021	18.23	**2022**	2025	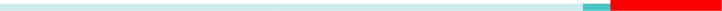
Woon EV, 2022, HUM REPROD UPDATE, V28, P548, DOI 10.1093/humupd/dmac006, DOI	2022	17.28	**2023**	2025	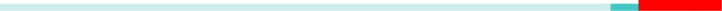
Atik RB, 2022, HUM REPROD OPEN, V2023, P0, DOI 10.1093/hropen/hoad002, DOI	2022	25.62	**2024**	2025	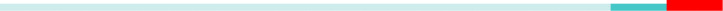

Bold values in the Begin column indicate the start year of the citation burst for each reference.

The publication with the highest citation burst is “Miscarriage matters: the epidemiological, physical, psychological, and economic costs of early pregnancy loss,” which remained in a burst state from 2022 to 2025 and drew attention for its broad summary of the epidemiological, clinical, and psychological burden of miscarriage. The “ESHRE guideline: recurrent pregnancy loss,” with a burst period from 2020 to 2025, reflected the growing influence of evidence-based clinical guidance in this field. The third-ranked article, titled “Recurrent pregnancy loss,” also remained in burst status from 2022 to 2025, highlighting sustained attention to the definition, etiology, and management of RPL. As of 2025, 73 articles were in a state of citation burst. The top 20 of these, ranked by burst strength, are listed in **Table 4**. This group comprised 11 review articles and 9 original articles.

**Table 4:** The references with citation bursts from beginning to 2025.

Begin	End	Strength	Year	Type	Title
2020	2025	48.98	2018	Review	Miscarriage matters: the epidemiological, physical, psychological, and economic costs of early pregnancy loss
2022	2025	43.05	2020	Article	ESHRE guideline: recurrent pregnancy loss
2005	2025	31	2004	Review	Recurrent pregnancy loss
2005	2025	27.91	2005	Article	Abnormal T-cell reactivity against paternal antigens in spontaneous abortion: adoptive transfer of pregnancy-induced CD4+CD25+ T regulatory cells prevents fetal rejection in a murine abortion model
2019	2025	26.87	2018	Article	Single-cell reconstruction of the early maternal-fetal interface in humans
2010	2025	23.92	2010	Article	Increased prevalence of T helper 17 (Th17) cells in peripheral blood and decidua in unexplained recurrent spontaneous abortion patients
2021	2025	23.83	2020	Review	T Helper (Th) Cell Profiles in Pregnancy and Recurrent Pregnancy Losses: Th1/Th2/Th9/Th17/Th22/Tfh Cells
2021	2025	22.92	2019	Review	Dynamic Function and Composition Changes of Immune Cells During Normal and Pathological Pregnancy at the Maternal-Fetal Interface
2011	2025	22.65	2010	Review	Th1/Th2/Th17 and regulatory T-cell paradigm in pregnancy
2007	2025	21.9	2006	Article	Decidual NK cells regulate key developmental processes at the human fetal-maternal interface
2018	2025	21.36	2017	Article	The unique immunological and microbial aspects of pregnancy
2013	2025	20.54	2012	Article	Extrathymic generation of regulatory T cells in placental mammals mitigates maternal-fetal conflict
2014	2025	20.2	2013	Review	Immunology of the maternal-fetal interface
2005	2025	19.89	2004	Article	Decidual and peripheral blood CD4+CD25+ regulatory T cells in early pregnancy subjects and spontaneous abortion cases
2010	2025	19.33	2009	Review	Regulatory T-cells and immune tolerance in pregnancy: a new target for infertility treatment?
2020	2025	19.15	2018	Article	Defective production of both leukemia inhibitory factor and type 2 T-helper cytokines by decidual T cells in unexplained recurrent abortions
2000	2025	18.94	1998	Review	Th1-type immunity is incompatible with successful pregnancy
2000	2025	18.94	1997	Review	Definitions of infertility and recurrent pregnancy loss: a committee opinion
2021	2025	18.54	2020	Review	The Role of Immune Cells in Recurrent Spontaneous Abortion
2022	2025	18.23	2021	Review	Immune responses at the maternal-fetal interface

### 3.3 Emerging trends and new developments

#### 3.3.1 The temporal variation of keyword clusters

**Figure 7:**
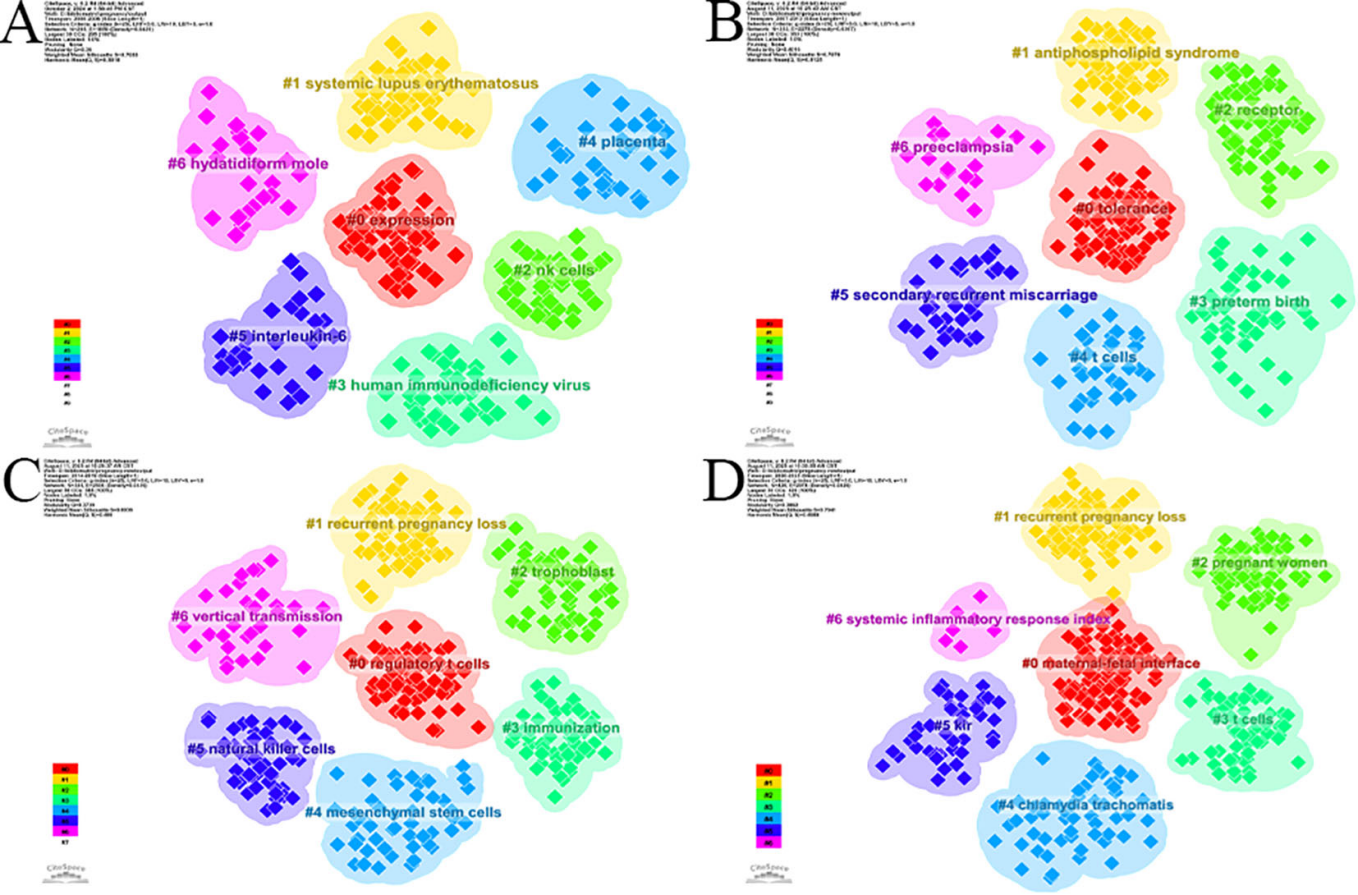
Evolution of keyword clusters in spontaneous abortion research across four time periods. Snapshots of thematic clusters derived from keyword co-occurrence analysis are shown for **(A)** 2000-2006, **(B)** 2007-2013, **(C)** 2014-2019, and **(D)** 2020-2025. Each color represents a distinct conceptual cluster. The visualization demonstrates the field’s progression from broad immunological concepts to a refined focus on the decidual microenvironment, specific pathologies, and clinical outcomes.

Keyword clustering helped to delineate the hotspot sub-domains within immunology and spontaneous abortion research. We divided the 25-year period into four phases, with keyword cluster snapshots generated for each (**Figure 7**). The first phase (2000–2006), yielded primary clusters including #0 expression, #1 systemic lupus erythematosus, and #2 nk cells (**Figure 7A**). The second phase (2007–2013) produced clusters such as #0 tolerance, #1 antiphospholipid syndrome, and #2 receptor (**Figure 7B**). The third phase (2014–2019) was dominated by clusters like #0 regulatory t cells, #1 recurrent pregnancy loss, and #2 trophoblast (**Figure 7C**). The final phase (2020–2025) generated clusters including #0 maternal-fetal interface, #1 recurrent pregnancy loss, and #2 pregnant women (**Figure 7D**).

Compared to the preceding 15 years, while classic research themes such as “human immunodeficiency virus” and “interleukin-6” remain relevant, emerging research clusters have garnered significantly more attention. These include #0 maternal-fetal interface, #1 recurrent pregnancy loss, #3 t cells, #4 chlamydia trachomatis, #5 kir, and #6 systemic inflammatory response index. Compared with the preceding periods, the most recent stage showed greater attention to clusters such as maternal-fetal interface, recurrent pregnancy loss, T cells, Chlamydia trachomatis, KIR, and systemic inflammatory response index. The representative keywords for these recent clusters are detailed in **Supplementary Table 3**.

#### 3.3.2 The keyword alluvial flow visualization

**Figure 8:**
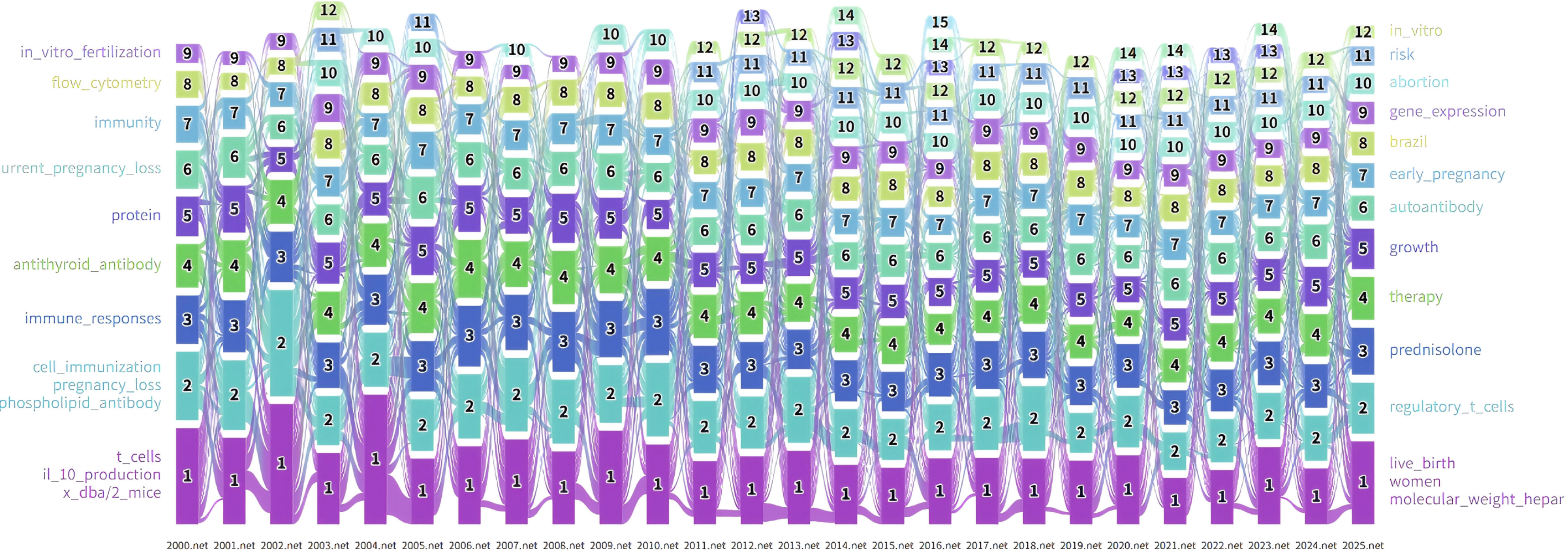
Alluvial flow map of keyword evolution in spontaneous abortion research (2000–2025). The map visualizes the rise, fall, and merging of thematic research modules over time. The X-axis represents the timeline divided into slices, and the Y-axis represents different thematic modules. The width of the streams corresponds to the number of keywords within a module, illustrating how research focus has shifted from general immunology to specific cellular pathways and clinical phenotypes over the 25-year period.

As illustrated in the alluvial diagram (**Figure 8**), related keywords were grouped into specific research modules. Over time, these modules differentiated or merged as keywords were reconfigured. Some modules showed substantial continuity across periods, whereas others gradually weakened or developed into new themes. The top five modules by keyword flow for each year are listed in **Supplementary Table 4**.

**Figure 9:**
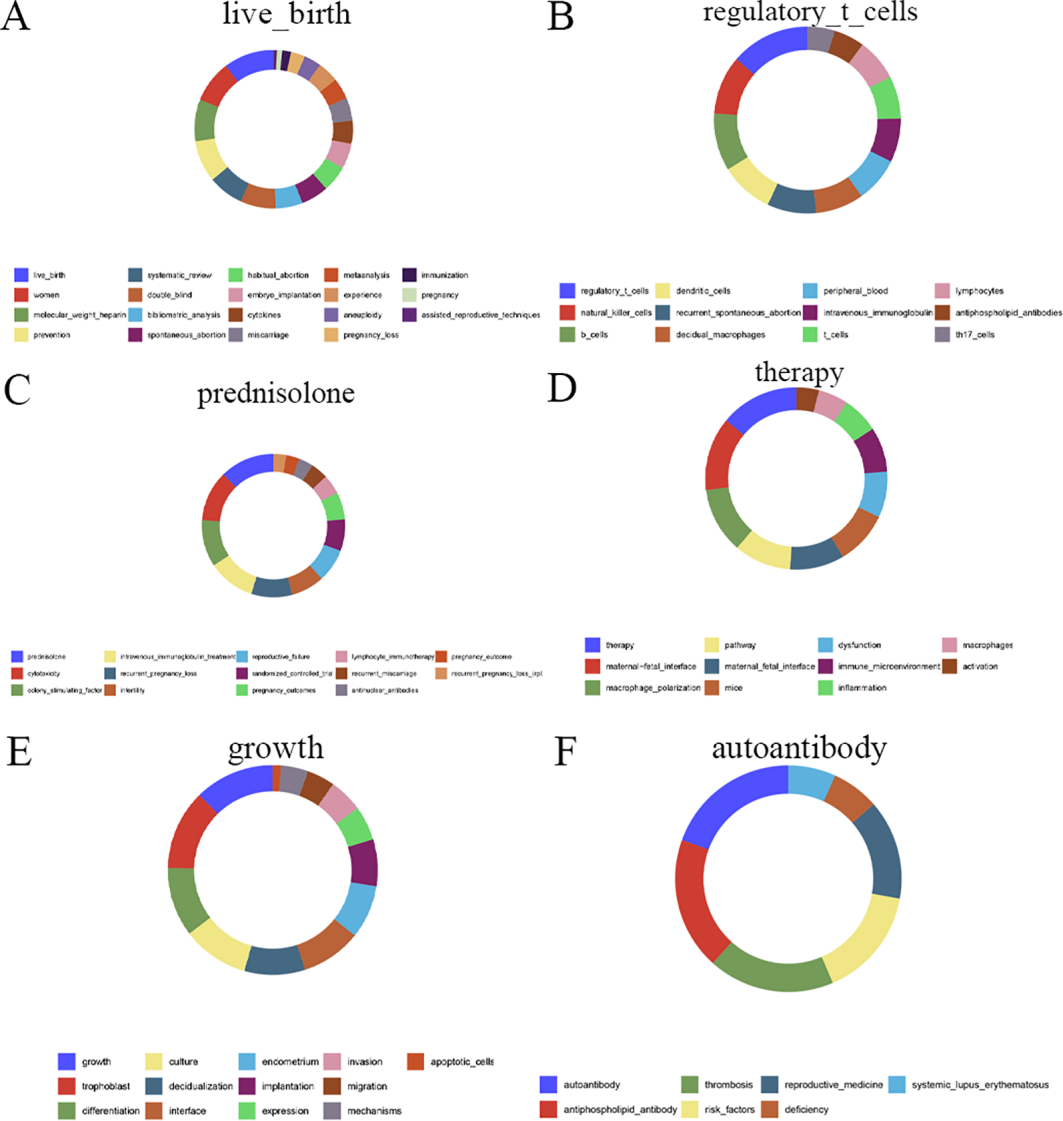
Keyword clusters of the top six emerging research modules in miscarriage. The modules represent the most active and distinct thematic sub-fields identified through clustering analysis. **(A)** Clinical management and outcomes. **(B)** Immune cell populations. **(C)** Immunomodulatory treatments. **(D)** Functional immunology of the maternal-fetal interface. **(E)** Trophoblast biology and implantation. **(F)** Autoimmunity and risk factors. These clusters highlight the field’s dominant focus on immunological mechanisms and their clinical translation.

Notably, the keywords forming Module 1 in 2025 constituted the largest and most persistent module in the recent stage (marked in red). **Figure 9** visualizes the keyword composition of the top six modules for 2025. Module 1, designated “live_birth,” encompasses 19 keywords such as “women,” “molecular_weight_heparin,” and “prevention” (**Figure 9A**). Module 2, “regulatory_t_cells,” includes 12 keywords like “natural_killer_cells,” “b_cells,” and “dendritic_cells” (**Figure 9B**). Module 3, “therapy,” contains 11 keywords including “maternal-fetal_interface,” “macrophage_polarization,” and “pathway” (**Figure 9C**). Module 4, “prednisolone,” comprises 14 keywords such as “cytotoxicity,” “colony_stimulating_factor,” and “intravenous_immunoglobulin_treatment” (**Figure 9D**). Module 5, “growth,” includes 13 keywords like “trophoblast,” “differentiation,” and “culture” (**Figure 9E**). Finally, Module 6, “autoantibody,” contains 7 keywords, including “antiphospholipid_antibody” and “thrombosis” (**Figure 9F**). These modules may reflect areas of sustained recent attention and potentially emerging thematic directions.

#### 3.3.3 The timeline visualization of references

A timeline visualization of co-cited references can effectively distinguish between emerging, classic, and relatively obsolete research topics. The timeline for this field is composed of 17 distinct clusters arranged by size (**Figure 1A**). The keyword co-occurrence network exhibited a Modularity Q value of 0.7622 and a Weighted Mean Silhouette S value of 0.8973, confirming that the identified clusters were both structurally significant and highly homogeneous.

Among these, clusters #1 tolerance, #5 chromosomal instability, #7 myeloid-derived suppressor cells, #9 influenza, and #13 antiphospholipid syndrome represent classic topics. While not necessarily at the cutting edge, they are deeply interconnected with other research areas. In contrast, clusters #3 md-1, #6 systemic lupus erythematosus, #8 natural killer cells, #10 cytokines, #14 thyroid autoimmunity, #15 il-11, and #16 tumor marker appear to be relatively outdated topics, showing minimal links to other clusters and little recent development along their own timelines. Finally, clusters #0 recurrent pregnancy loss, #2 maternal-fetal interface, #4 intralipid, #11 chronic endometritis, and #12 covid-19 are identified as emerging topics. Their continuous activity from their inception to the present suggests that they remain active and potentially important areas of ongoing research. **Supplementary Table 5** provides further details on these emerging clusters.

Several classic papers (large nodes with red rings) have played a pivotal role in driving the development of these sub-fields (**Figure 1B**). For example, Wang WJ’s 2018 review in cluster #0, which comprehensively outlined the roles of various T helper cell subsets in both normal and pathological pregnancies, has been co-cited 68 times. In cluster #2, the 2018 paper by Vento-Tormo R, which presented a single-cell reconstruction of the early maternal-fetal interface, identified novel cell subtypes and their interactions essential for successful placentation (co-cited 73 times). A key paper in cluster #4 identified distinct Th17/Treg cell patterns in women with unexplained recurrent spontaneous abortion, linking an imbalanced maternal-fetal immune tolerance to the condition (co-cited 37 times). A central work in the emerging cluster #11 demonstrated that chronic endometritis is associated with elevated immune cell infiltration in the endometrium of patients with recurrent reproductive failure. Finally, in cluster #12, the living systematic review by Allotey J established that pregnant women with COVID-19 face significantly higher risks of severe outcomes, including maternal death, intensive care unit (ICU) admission, and preterm birth, compared to non-pregnant counterparts.

The recent citation distribution of these five influential articles (**Figure 1C**) indicates that they remain highly visible within the recent literature.

## 4 Discussion

This study presents the first data-driven, panoramic dissection of the global research landscape in the immunology of spontaneous abortion over the last quarter-century. By applying scientometric analysis to a corpus of 4,495 publications, we have moved beyond narrative review to quantitatively map the field’s intellectual architecture, collaboration dynamics, and thematic evolution. Our analysis reveals a profound paradigm shift, away from systemic immunological descriptions and towards a high-resolution, mechanistic exploration of the local maternal-fetal interface. We have charted a clear evolutionary trajectory from foundational concepts, such as immune tolerance and antiphospholipid syndrome, to the current major areas of research, which is dominated by emerging frontiers including the maternal-fetal interface, CE, and the immunological sequelae of COVID-19. While these shifts are indicative of significant progress, a critical examination of the underlying structural and translational factors that have shaped—and constrained—the trajectory of this field is essential for contextualizing our findings and charting future directions.

Our finding of an exponential growth in publication output since 2008 directly correlates with technological revolutions that have reshaped reproductive immunology (34). The advent and subsequent refinement of high-throughput technologies, particularly single-cell and spatial transcriptomics, have empowered researchers to deconstruct the cellular composition and spatial organization of the maternal-fetal interface with unparalleled resolution (35, 36). This technological leap is the primary driver behind “maternal-fetal interface” emerging as the keyword with the highest burst strength and centrality in our analysis. This trend reflects a crucial scientific consensus: the outcome of pregnancy is determined not by systemic immunity alone, but by the dynamic, localized crosstalk between maternal decidual cells and fetal trophoblasts (37). Recent landmark studies, for example, have precisely mapped the transcriptional identities and functional plasticity of distinct decidual NK cell, macrophage, and T cell subsets in early pregnancy, revealing significant dysregulation in patients with RPL (38, 39). This transition from a relatively poorly characterized concept to a molecularly defined microenvironment has become an important basis for the field’s progression toward precision medicine.

Intricately linked to the focus on the interface is the strong emergence of CEand RIFas high-strength keywords. This signifies another critical evolution in the research paradigm: a temporal shift of focus from the post-implantation loss event to the pre-conceptional endometrial microenvironment that governs receptivity (40). Long considered a secondary gynecological issue, CE, a persistent, low-grade inflammation often driven by microbial dysbiosis, is now recognized as a significant and treatable cause of RPL and RIF (41). A growing body of evidence demonstrates that CE disrupts the immune tolerance required for implantation by altering the local immune cell landscape, including promoting plasma cell infiltration and aberrant B cell activation (42, 43). Our analysis quantitatively captures this sharp rise in clinical and research interest, which underscores the uterus’s role as a critical immune organ and has opened new diagnostic and therapeutic avenues based on endometrial microbiome and immunobiome profiling (44, 45).

At the level of specific immune mechanisms, our alluvial flow diagrams and cluster analyses reveal a sophisticated thematic evolution. Early research was heavily anchored in the classic Th1/Th2 cytokine balance model (46). However, as the immunological toolkit expanded, this paradigm proved insufficient to explain the complexities of gestational immune regulation. Our data show a clear progression toward a multidimensional, interactive network encompassing Tregs, Th17 cells, follicular helper T (Tfh) cells, and B cells (47). The module related to “regulatory_t_cells,” in particular, demonstrates remarkable persistence and evolutionary potential in the alluvial flow, a finding that aligns perfectly with functional studies. The stability and functional diversity of Tregs within the decidua are now understood to be critical not only for suppressing inflammation but also for promoting angiogenesis and tissue remodeling (48). Consequently, a numerical or functional deficit in Tregs, especially their conversion to a pro-inflammatory phenotype, is now recognized as a key pathogenic event in RPL (49). Therefore, understanding and therapeutically targeting the differentiation, migration, and function of Tregs represents a core objective of modern immunotherapeutic strategies.

Perhaps most revealingly, our analysis captured the immediate impact of major public health events on the field’s research agenda, exemplified by the rapid emergence of the “COVID-19” cluster. This demonstrates that the immunology of spontaneous abortion is not an isolated discipline but is dynamically intertwined with global health challenges (50). Research into the effects of SARS-CoV-2 infection on pregnancy has not only illuminated how viral infections can compromise the maternal-fetal interface—through mechanisms such as cytokine storms, complement activation, and angiopathy—leading to increased risks of miscarriage and preterm birth (51, 52), but it has also provided a unique model for studying how acute systemic inflammation disrupts established immune tolerance in pregnancy (53). This has prompted the community to re-evaluate the role of other pathogens (e.g., cytomegalovirus, toxoplasma) and non-infectious systemic inflammatory states (e.g., autoimmune diseases) in the etiology of RPL, highlighting the critical importance of infection control during gestation.

Notwithstanding the scientific progress documented above, a critical observation from our study is the striking geographic concentration of research output. The dominance of China and the USA in terms of publication volume, while reflecting substantial research infrastructure and investment, raises important questions about the global representativeness of the current evidence base. This geographic skew is not merely a bibliometric artifact but carries substantive implications for the field. Studies from low- and middle-income countries, where the burden of infectious diseases, nutritional deficiencies, and limited healthcare access may shape distinct immunological profiles and pregnancy outcomes, remain markedly underrepresented (54). Consequently, the mechanistic insights and therapeutic priorities emerging from high-volume regions may not be universally applicable, potentially narrowing our understanding of spontaneous abortion’s heterogeneous etiology across diverse populations. A more inclusive approach to global collaboration and research capacity-building is essential for ensuring that the intellectual architecture of the field reflects a truly global perspective (55).

Furthermore, the high publication output from leading countries does not automatically equate to proportional research impact. While citation counts and publication frequency are commonly employed as proxies for scientific influence, they capture neither the qualitative rigor of research nor its translational utility. Citation metrics can be inflated by self-citation practices, preferential citation within regional networks, or a tendency to publish in high-impact factor journals that may prioritize novelty over reproducibility (56). Our findings thus underscore the need for the field to move beyond volume-based metrics toward more nuanced assessments of research quality. Initiatives promoting open science, data sharing, preregistration of clinical studies, and replication efforts would strengthen the evidentiary foundation upon which clinical decisions are ultimately based.

Perhaps the most consequential gap illuminated by our analysis is the persistent disconnect between accelerating mechanistic discovery and the slow pace of clinical translation. While the thematic shifts toward the maternal-fetal interface, CE, and Treg biology represent genuine advances in fundamental understanding, these insights have not yet crystallized into effective, evidence-based immunotherapies for RPL. The convergence of keyword modules such as “therapy,” “live_birth,” “prednisolone,” and “intravenous_immunoglobulin_treatment” in the alluvial flow map reflects the intense drive to translate mechanistic discoveries into clinical practice. However, immunotherapy in this field has long been controversial, with many conventional treatments lacking high-quality, evidence-based support, leading to significant heterogeneity in clinical practice (57, 58). This is largely due to a historical “one-size-fits-all” approach that ignores the highly heterogeneous immunopathology of RPL (59, 60). The future of therapy lies in precise patient stratification based on “immune endotyping” (61, 62). This could involve using flow cytometry or mass cytometry (CyTOF) to profile immune cell subsets in peripheral blood or endometrial biopsies, or using serological assays to identify specific cytokine or autoantibody profiles, thereby classifying patients into distinct subtypes, such as Th1/Th17-dominant, NK cell cytotoxicity-driven, or autoimmune-mediated RPL (63). This would enable the rational design of targeted therapies, such as using TNF-α inhibitors or hydroxychloroquine for Th1/Th17-skewed patients, exploring low-dose IL-2 or adoptive Treg transfer for those with Treg deficiencies, or employing more specific immunosuppressants for patients with defined autoantibodies. However, realizing this vision requires not only continued mechanistic discovery but also rigorous prospective validation of biomarkers, standardization of diagnostic platforms, and, critically, well-designed clinical trials that embrace heterogeneity rather than ignoring it.

Finally, our analysis reveals a notable disconnect between rapidly evolving research fronts and the comparatively static landscape of clinical guideline development. The lag between scientific evidence and guideline incorporation is not unique to reproductive immunology, but it is particularly consequential given the vulnerability of the patient population and the emotional and physical toll of RPL. The lack of consensus across international guidelines regarding the role of immunotherapy in RPL reflects both the limitations of the existing evidence base and the challenges of synthesizing heterogeneous, often underpowered studies. While scientometric methods provide valuable insights into research trends and emerging priorities, these findings must be effectively translated into clinical practice through improved collaboration between researchers, clinicians, and guideline development bodies. This will require not only high-quality evidence from well-designed trials but also frameworks for integrating mechanistic insights with patient-reported outcomes and health economic considerations.

In conclusion, this study provides a comprehensive overview of the intellectual evolution in the immunology of spontaneous abortion over the past quarter-century. The field has undergone a remarkable transformation, progressing from systemic immunological descriptions to high-resolution mechanistic explorations of the maternal-fetal interface, driven by technological innovation and an increasingly sophisticated understanding of immune regulation at the maternal-fetal frontier. The rapid growth in research output and the identification of emerging frontiers such as CE and COVID-19’s impact on pregnancy represent genuine scientific progress. However, significant challenges remain. Addressing geographic publication imbalances, improving the quality and reproducibility of research, bridging the persistent gap between basic immunology and clinical practice, and aligning research priorities with the rigorous demands of evidence-based guideline development are essential tasks for the coming decade. By confronting these challenges with the same rigor that has characterized the field’s scientific evolution, reproductive immunology can fulfill its promise of delivering effective, personalized treatments for RPL and improving outcomes for women worldwide.

## 5 Limitations

Despite the panoramic insights provided by this 25-year analysis, several inherent limitations must be acknowledged.

First, the exclusive reliance on the Web of Science Core Collection introduces inherent database selection bias. While WoSCC provides the most standardized citation metadata required for our specific software tools, it does not fully capture all literature indexed in Scopus, PubMed/MEDLINE, or Embase. Consequently, relevant articles exclusive to those platforms, as well as non-English regional literature, may have been omitted. Furthermore, all reported citation metrics—including burst strengths and co-citation frequencies—are strictly dependent on WoSCC indexing and should be interpreted as relative metrics within this ecosystem rather than absolute measures of global impact.

Second, the citation time-lag effect is unavoidable. Scientometric metrics favor older publications that have had sufficient time to accumulate citations; consequently, cutting-edge discoveries from 2024–2025 may appear less influential in terms of total citation counts, even if they exhibit high citation bursts.

Third, clustering results are subject to algorithmic parameter dependency. The thematic boundaries of modules like “maternal-fetal interface” are influenced by CiteSpace’s time-slicing intervals and labeling algorithms. While our validation metrics (Q and S values) confirm structural robustness, alternative parameter configurations might yield slightly different cluster resolutions.

Finally, bibliometric analysis measures scholarly impact and visibility rather than methodological quality. A high citation count signifies that a paper is influential in the academic dialogue, but it does not inherently guarantee superior experimental design or clinical evidence strength. Therefore, the hotspots identified herein should be viewed as dominant trends in the scientific literature rather than a direct assessment of clinical truth.

## Data Availability

The original contributions presented in the study are included in the article/**Supplementary Material**. Further inquiries can be directed to the corresponding author.
