# Supplementary material for: Global research trends and future frontiers in the immunology of spontaneous abortion: a 25-year scientometric analysis
Source: Front Immunol. 2026 Apr 21;17:1753732. doi: 10.3389/fimmu.2026.1753732 (PMC13139909; doi:10.3389/fimmu.2026.1753732)
Supplement: Supplementary file 1 [file Table1.docx]

**Supplementary materials**

Table S1. The top 10 countries, institutions and authors for frequency of co-occurrence.

| **CoF** | **Year** | **Country** |  | **CoF** | **Year** | **Institution** |  | **CoF** | **Citation** | **Author** |
| --- | --- | --- | --- | --- | --- | --- | --- | --- | --- | --- |
| 1079 | 2001 | CHINA |  | 120 | 2005 | Fudan University |  | 40 | 2230 | kwak-kim, joanne |
| 924 | 2000 | USA |  | 94 | 2005 | Shanghai Jiao Tong University |  | 32 | 1284 | li, da-jin |
| 344 | 2000 | ENGLAND |  | 71 | 2000 | Harvard University |  | 30 | 753 | li, ming-qing |
| 247 | 2000 | GERMANY |  | 70 | 2001 | Institut National de la Sante et de la Recherche Medicale (Inserm) |  | 29 | 553 | jeschke, udo |
| 235 | 2000 | ITALY |  | 67 | 2000 | Assistance Publique Hopitaux Paris (APHP) |  | 29 | 3196 | saito, shigeru |
| 225 | 2000 | JAPAN |  | 67 | 2002 | University of London |  | 28 | 507 | yang, jing |
| 160 | 2004 | IRAN |  | 67 | 2002 | University of Copenhagen |  | 26 | 874 | du, meirong |
| 158 | 2001 | CANADA |  | 65 | 2000 | University of California System |  | 25 | 819 | yousefi, mehdi |
| 144 | 2000 | INDIA |  | 64 | 2002 | Copenhagen University Hospital |  | 25 | 501 | zhang, yan |
| 142 | 2000 | FRANCE |  | 58 | 2006 | Centers for Disease Control & Prevention - USA |  | 23 | 716 | clark, david a. |

CoF: Co-occurrence frequency. Year:Year of first collaboration appearance

Table S2. The top 20 subject categories and keywords burst with a burst period from beginning to 2024.

| **Subject category bursts** | | | | | **Keywords bursts** | | | | |
| --- | --- | --- | --- | --- | --- | --- | --- | --- | --- |
| Begin | End | Strength | Year | Entity | Begin | End | Strength | Year | Entity |
| 2021 | 2025 | 15.75 | 2000 | MEDICINE, GENERAL & INTERNAL | 2020 | 2025 | 15.87 | 2019 | recurrent implantation failure |
| 2021 | 2025 | 12.49 | 2010 | CHEMISTRY, MULTIDISCIPLINARY | 2020 | 2025 | 13.4 | 2015 | chronic endometritis |
| 2021 | 2025 | 8.82 | 2000 | BIOCHEMISTRY & MOLECULAR BIOLOGY | 2019 | 2025 | 10.35 | 2019 | maternal-fetal interface |
| 2024 | 2025 | 3.87 | 2013 | PLANT SCIENCES | 2021 | 2025 | 9.55 | 2019 | migration |
| 2024 | 2025 | 3.77 | 2004 | CHEMISTRY, MEDICINAL | 2022 | 2025 | 9.22 | 2017 | immune cells |
| 2024 | 2025 | 3.32 | 2002 | INTEGRATIVE & COMPLEMENTARY MEDICINE | 2021 | 2025 | 9.14 | 2013 | outcm |
| 2021 | 2025 | 1.95 | 2015 | ENGINEERING, BIOMEDICAL | 2021 | 2025 | 8.72 | 2021 | live birth rate |
| 2021 | 2025 | 1.95 | 2018 | CELL & TISSUE ENGINEERING | 2021 | 2025 | 8.69 | 2015 | pregnancy complications |
| 2024 | 2025 | 1.77 | 2001 | MICROBIOLOGY | 2023 | 2025 | 8.53 | 2020 | pregnancy outcomes |
| 2020 | 2025 | 1.76 | 2014 | PRIMARY HEALTH CARE | 2024 | 2025 | 7.1 | 2000 | recurrent pregnancy loss |
| 2022 | 2025 | 1.73 | 2016 | MATERIALS SCIENCE, BIOMATERIALS | 2021 | 2025 | 6.74 | 2018 | pathway |
| 2022 | 2025 | 1.53 | 2018 | NANOSCIENCE & NANOTECHNOLOGY | 2022 | 2025 | 6.55 | 2004 | embryo implantation |
| 2024 | 2025 | 1.35 | 2003 | ENVIRONMENTAL SCIENCES | 2021 | 2025 | 5.95 | 2016 | promotes |
| 2024 | 2025 | 1.18 | 2020 | POLYMER SCIENCE | 2024 | 2025 | 5.82 | 2019 | live birth |
| 2024 | 2025 | 1.18 | 2013 | ENGINEERING, ENVIRONMENTAL | 2021 | 2025 | 5.38 | 2012 | endometrial receptivity |
| 2024 | 2025 | 1.16 | 2024 | MATERIALS SCIENCE, MULTIDISCIPLINARY | 2023 | 2025 | 5.35 | 2008 | thyroid autoimmunity |
| 2024 | 2025 | 1.11 | 2015 | CHEMISTRY, APPLIED | 2023 | 2025 | 5.26 | 2019 | age |
| 2024 | 2025 | 0.95 | 2000 | PSYCHIATRY | 2022 | 2025 | 5.25 | 2022 | immunogenicity |
| 2023 | 2025 | 0.75 | 2001 | BIOPHYSICS | 2021 | 2025 | 5.22 | 2000 | reproductive immunology |
| 2024 | 2025 | 0.62 | 2024 | SOCIAL WORK | 2023 | 2025 | 5.14 | 2023 | macrophage polarization |

Bigin: the burst’ beginning year, End: the burst’ ending year, Strength: the burst’ strength index, Year: the first appearance time, Entity: the term.

Table S3. Summary of keyword clusters for the most recent stage(2020-2025).

| **ClusterID** | **Size** | **Silhouette** | **Average Year** | **Label (LLR)** | **Representative keywords** |
| --- | --- | --- | --- | --- | --- |
| 0 | 101 | 0.611 | 2021 | maternal-fetal interface | recurrent spontaneous abortion; peripheral blood; pregnancy complications; feto-maternal cross-talk; endometrial stromal cells \| maternal-fetal interface; endometrial stromal cells; animal models; spontaneous abortion; decidual macrophages |
| 1 | 92 | 0.727 | 2021 | recurrent pregnancy loss | recurrent pregnancy loss; chronic endometritis; transcription factor; hpv vaccine; review recurrent pregnancy loss \| recurrent miscarriage; genetic factors; based medicine; treatment strategy; nk cell activity |
| 2 | 68 | 0.746 | 2021 | pregnant women | spontaneous abortion; maternal immune tolerance; induced abortion; reproductive immunology; young women \| pregnant women; vaccine safety; influenza vaccine; adverse birth outcomes; prospective cohort study |
| 3 | 58 | 0.745 | 2021 | t cells | recurrent spontaneous abortion; costimulatory molecules; unexplained recurrent miscarriage; transcription factor; spontaneous abortion \| recurrent pregnancy loss; sildenafil citrate; unexplained recurrent miscarriage; transcription factor; spontaneous abortion |
| 4 | 58 | 0.695 | 2021 | chlamydia trachomatis | endometrial receptivity; embryo implantation; pregnancy loss; maternal immunisation; immunoglobulin g \| recurrent spontaneous abortion; recurrent implantation failure; premature ovarian failure; human amniotic epithelial cells; canid alphaherpesvirus |
| 5 | 42 | 0.724 | 2021 | kir | recurrent miscarriage; recurrent implantation failure; reproductive failure; immune cells; tet enzymes \| recurrent miscarriages; intravenous immunoglobulin; unexplained recurrent miscarriage; chromosomal microarray; spontaneous abortion |
| 6 | 7 | 0.964 | 2024 | systemic inflammatory response index | systemic immune- inflammatory index; systemic inflammatory response index; early pregnancy loss; inflammatory biomarkers; mean platelet volume \| recurrent pregnancy loss; internal medicine; placental pathology; antiphospholipid antibody syndrome; systemic immune-inflammation index |

Size: the number of articles in each cluster; Silhouette: the average contour value of clustering, it is generally believed that the clustering category with S > 0.5 is reasonable, and S > 0.7 means that the clustering is convincing; LLR: Log-likelihood ratio.

Table S4. The most trafficked keyword for the top five modules each year.

| **Year** | **2000** | **2001** | **2002** | **2003** | **2004** | **2005** | **2006** | **2007** | **2008** | **2009** |
| --- | --- | --- | --- | --- | --- | --- | --- | --- | --- | --- |
| Total modules | 7 | 6 | 4 | 6 | 7 | 6 | 9 | 9 | 11 | 9 |
| module1 | t_cells | reproductive_failure | repeated_fetal_losses | gestational_age | lupus_anticoagulant | indoleamine_2 | interleukin_1_receptor_antagonist | human_pregnancy | definite_antiphospholipid_syndrome | endometriosis |
| module2 | cell_immunization | leukocyte_immunization | nk_cells | large_granular_lymphocytes | abortion | menstrual_cycle | paternal_lymphocytes | hydatidiform_mole | dydrogesterone_supplementation | immunity |
| module3 | immune_responses | receptors | randomized_controlled_trial | gamma | fetomaternal_interface | major_histocompatibility_complex | normal_human_pregnancy | molecular_weight_heparin | cytotoxicity | immunoglobulin_like_receptors |
| module4 | reproductive_failure | killer_cells | recurrent_abortion | lupus_anticoagulants | human_endometrium | intravenous_immunoglobulin_therapy | molecular_weight_heparin | experimental_antiphospholipid_syndrome | complement_activation | immunological_self_tolerance |
| module5 | necrosis_factor_alpha | molecular_weight_heparin | abnormality | killer_cell_activity | factor_v_leiden | maternal_fetal_interface | general_practice | double_blind | colony_stimulating_factor | factor_kappa_b |
| **Year** | **2010** | **2011** | **2012** | **2013** | **2014** | **2015** | **2016** | **2017** | **2018** | **2019** |
| Total modules | 8 | 13 | 10 | 12 | 11 | 12 | 13 | 14 | 9 | 11 |
| module1 | growth_restriction | controlled_trial | endometrial_receptivity | chromosomal_instability | antiprothrombin_antibody | antithrombotic_therapy | allograft_rejection | delivery | nf_kappa_b | adhesion |
| module2 | heparin | birth_weight | fetal_tolerance | autoimmunity | unexplained_recurrent_spontaneous_abortion | unexplained_recurrent_spontaneous_abortion | randomized_controlled_trial | systematic_review | vertical_transmission | advisory_committee |
| module3 | factor_alpha | antigens | g_messenger_rna | circulating_procoagulant_microparticles | metrial_gland_cells | transplant_recipients | pregnancy_outcome | d_deficiency | anticardiolipin_antibody | molecular_weight_heparin |
| module4 | fetomaternal_interface | angiogenic_factors | fetal | congenital_uterine_anomaly | autoimmunity | fetal_death | animal_model | endometrium | pregnancy_complications | genotype |
| module5 | cd4(+)cd25(+) | unk_cells | nk_cell | transcription_factor | female_sex_hormones | bone_marrow_transplantation | uterine_nk_cells | cytotoxicity | transcription_factor_foxp3 | adoptive_transfer |
| **Year** | 2020 | 2021 | 2022 | 2023 | 2024 | 2025 |  |  |  |  |
| Total modules | 15 | 16 | 13 | 14 | 12 | 12 |  |  |  |  |
| module1 | immunization_practices | identification | obstetric_antiphospholipid_syndrome | tumor_necrosis_factor | bioinformatics_analysis | live_birth |  |  |  |  |
| module2 | polymerase_chain_reaction | immunohistochemistry | pregnancy_registry | macrophage_polarization | animal_model | regulatory_t_cells |  |  |  |  |
| module3 | markers | molecular_weight_heparin | infertile_women | unexplained_infertility | advisory_committee | prednisolone |  |  |  |  |
| module4 | immune_tolerance | implantation_failure | polarization | repeated_implantation_failure | subclinical_hypothyroidism | therapy |  |  |  |  |
| module5 | pd_1 | maternal_age | early_pregnancy_loss | decidual_nk_cells | immunoglobulin_like_receptors | growth |  |  |  |  |

Table S5. Summary of emerging topics.

| **ClusterID** | **Size** | **Silhouette** | **Average Year** | **Label (LLR)** | **Representative keywords** |
| --- | --- | --- | --- | --- | --- |
| 0 | 211 | 0.878 | 2020 | recurrent pregnancy loss | recurrent pregnancy loss; inhibitory factor; machine learning; high-dimensional weighted gene co-expression network analysis; decidual natural killer cells \| recurrent miscarriage; reproductive immunology; regulatory t cells; intravenous immunoglobulin; immunomodulatory therapy |
| 2 | 174 | 0.862 | 2018 | maternal-fetal interface | maternal-fetal interface; immune cells; gamma delta t cells; growth factor; unexplained recurrent miscarriage \| recurrent miscarriage; nk cells; maternal-foetal immunotolerance; nk cell-based immunotherapy; unexplained recurrent miscarriage |
| 4 | 141 | 0.856 | 2015 | intralipid | recurrent miscarriage; regulatory t cells; reproductive immunology; decidual immune cells; paternal immunotherapy \| recurrent pregnancy loss; t cells; anticardiolipin antibody; negative apla; reproductive technology |
| 11 | 29 | 0.993 | 2018 | chronic endometritis | chronic endometritis; antibiotic treatment; live birth rate; miscarriage rate; spontaneous abortion \| recurrent pregnancy loss; implantation failure; early pregnancy loss; late pregnancy loss; endometritis |
| 12 | 25 | 0.995 | 2020 | covid-19 | vertical transmission; severe acute respiratory syndrome; preterm birth; pregnancy loss; nf-kappa b pathway \| first trimester; pregnancy vaccination; preterm birth; vaccine hesitancy; mrna vaccines |
